# Comparative Study on the Magnetic and Transport Properties of B-Site Ordered and Disordered CaCu_3_Fe_2_Os_2_O_12_

**DOI:** 10.1021/acs.inorgchem.2c03030

**Published:** 2022-10-10

**Authors:** Xiao Wang, Zhehong Liu, Hongshan Deng, Stefano Agrestini, Kai Chen, Jyh-Fu Lee, Hong-Ji Lin, Chien-Te Chen, Fadi Choueikani, Philippe Ohresser, Fabrice Wilhelm, Andrei Rogalev, Liu Hao Tjeng, Zhiwei Hu, Youwen Long

**Affiliations:** † Beijing National Laboratory for Condensed Matter Physics, Institute of Physics, Chinese Academy of Sciences, Beijing 100190, China; ‡ Max Planck Institute for Chemical Physics of Solids, Dresden 01187, Germany; § School of Physical Sciences, University of Chinese Academy of Sciences, Beijing 100049, China; ∥ ALBA Synchrotron Light Source, Cerdanyola del Vall′es, Barcelona E-08290, Spain; ⊥ National Synchrotron Radiation Research Center, Hsinchu 30076, Taiwan; # Synchrotron SOLEIL, L’Orme des Merisiers, Saint-Aubin, BP 48, Gif-sur-Yvette Cedex 91192, France; ∇ European Synchrotron Radiation Facility, 71 Avenue des Martyrs, Grenoble 38043, France; ○ Songshan Lake Materials Laboratory, Dongguan, Guangdong 523808, China

## Abstract

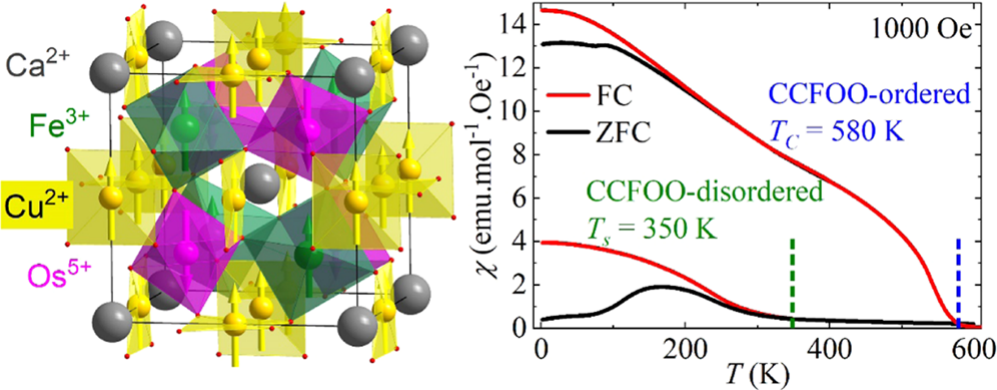

The B-site Fe/Os ordered and disordered quadruple perovskite oxides CaCu_3_Fe_2_Os_2_O_12_ were synthesized under different high-pressure and high-temperature conditions. The B-site ordered CaCu_3_Fe_2_Os_2_O_12_ is a system with a very high ferrimagnetic ordering temperature of 580 K having the Cu^2+^(↑)Fe^3+^(↑)Os^5+^(↓) charge and spin arrangement. In comparison, the highly disordered CaCu_3_Fe_2_Os_2_O_12_ has a reduced magnetic transition temperature of about 350 K. The Cu^2+^Fe^3+^Os^5+^ charge combination remains the same without any sign of changes in the valence state of the constituent ions. Although the average net moments of each sublattice are reduced, the average ferrimagnetic spin arrangement is unaltered. The robustness of the basic magnetic properties of CaCu_3_Fe_2_Os_2_O_12_ against site disorder may be taken as an indication of the tendency to maintain the short-range order of the atomic constituents.

## Abstract 2

The B-site Fe/Os ordered and disordered quadruple perovskite oxides CaCu_3_Fe_2_Os_2_O_12_ were synthesized at different high-pressure and high-temperature conditions. Both compounds form the Cu^2+^(↑)Fe^3+^(↑)Os^5+^(↓) valence and magnetic arrangement. The ordered perovskite experiences a long-range ferrimagnetic transition at 580 K on account of the strong Cu−Os coupling. However, the disordered one undergoes a spin-glass transition at 350 K because the Cu−Os coupling is disturbed by the disorder. The relative robustness of the magnetic properties against disorder may be taken as an indication of the presence of short-range order of the B-site cations.

## 1 Introduction

**Figure 1:**
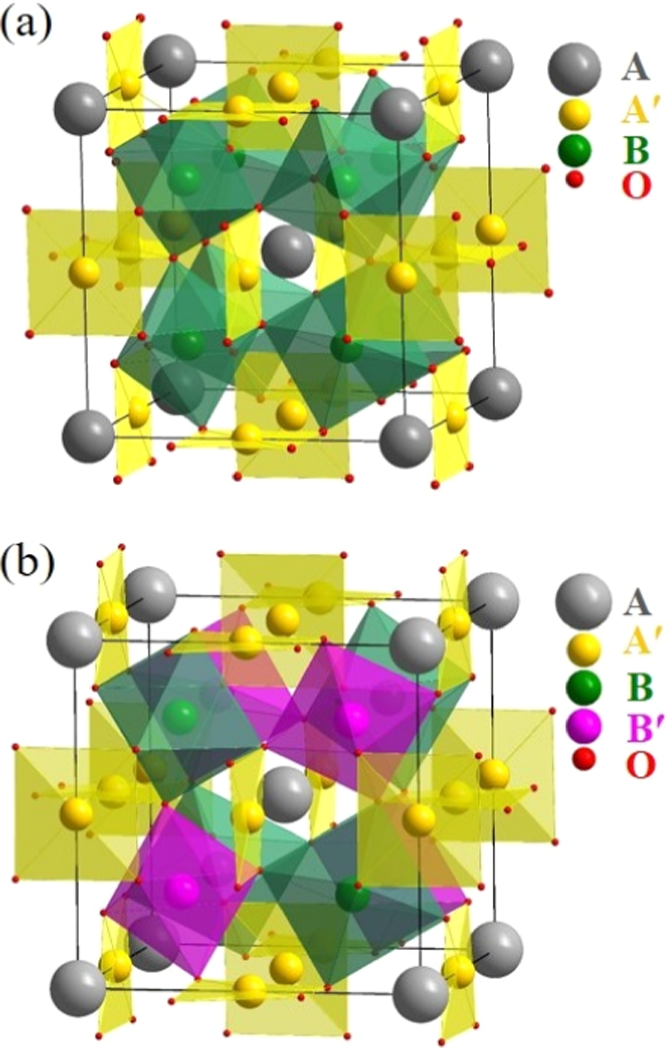
Schematic crystal structures of quadruple perovskite oxides (a) AA′_3_B_2_B′_2_O_12_ with the disordered B-site and (b) AA′_3_B_2_B′_2_O_12_ with the B/B′-site ordered in a rock-salt fashion.

Perovskite and perovskite-like compounds exhibit a wide variety of intriguing physical properties due to the high flexibility of crystal constructions and accommodating atoms in the structures.^1−8^ In a simple ABO_3_ perovskite, alkali, alkaline earth, and/or rare earth elements can reside at the A-site and transition metals usually occupy the B-site. For such a simple ABO_3_ perovskite, when three-quarters of the A-sites are replaced by transition metals and, simultaneously, half of the B-sites are substituted by another kind of transition metal, one may obtain a peculiar quadruple perovskite oxide with the chemical formula of AA′_3_B_2_B′_2_O_12_. Since the A′-site is occupied by a transition metal that is coordinated with four oxygens and forms a square-planar unit, the Jahn–Teller ions such as Cu^2+^ and Mn^3+^ are appropriate choices. The drastic distortion of the structure gives rise to an orderly distribution of the A- and A′-sites with a 1:3 ratio. However, because both B- and B′-sites accommodate transition metals and form an octahedron with six coordinated oxygens, these two sites can either be disordered (Figure 1a) or ordered in a rock-salt fashion (Figure 1b).^9,10^ In quadruple perovskite oxides, multiple transition metals reside at A′-, B-, and B′-sites, which will introduce plenty of novel magnetic and electric interaction pathways such as A′-B, A′-B′, and B-B′. As a consequence, quadruple perovskite oxides exhibit a series of interesting properties like intersite charge transfer,^1^ high-temperature half metallicity,^2−4^ cubic multiferroicity,^5−7^ and charge disproportionation.^8^

Recently, a new quadruple perovskite oxide CaCu_3_Fe_2_Os_2_O_12_ (CCFOO) with B-site ordered Fe/Os distribution was successfully synthesized.^11^ It possesses a very high ferrimagnetic Curie temperature *T*_C_ of 580 K and a considerable magnetic moment of 5.0 μ_B_/fu. The charge and spin configuration is Cu^2+^(↑)Fe^3+^(↑)Os^5+^(↓),^11^ quite analogous to some other isostructural systems^2,3,12^ and with also signs for the presence of orbital moments.^3,13^ It is worth noting that CCFOO was synthesized under the high-pressure and high-temperature (HPHT) method, which is able to stabilize the metastable phase at ambient pressure.^14−17^

We now would like to know what effect disorder could have on the magnetic properties of such a complex system as the quadruple perovskites. In La_2_MnCoO_6_, for example, the Mn–Co disorder leads not only to a lower magnetic ordering temperature but also to changes in the Mn and Co valence states.^18^ Such an order–disorder study for the quadruple perovskites is, however, not so straightforward. Ordering of the B- and B′-sites requires remarkable differences in ionic radii and valence states between the two different elements, and high pressures up to several GPa and a relatively lower temperature are required during the synthesis. However, the requirements for the disordered B-sites are almost the opposite, i.e., similar B/B′ ionic radii, relatively lower pressures, and higher temperatures are needed. Thus one has to carefully select the combination of B/B′ elements and adjust the synthesizing conditions to obtain both the B-site ordered and disordered perovskites.

In this study, we have successfully synthesized both the B-site (Fe/Os) ordered and disordered CCFOO, using different HPHT conditions. We have performed the element-specific X-ray absorption spectroscopy (XAS) and X-ray magnetic circular dichroism (XMCD) measurements^19−21^ to determine the magnitude and orientation of the magnetic moments on each atomic (Cu, Fe, and Os) constituent separately.

## 2 Experimental Section

High purity (>99.9%) powders of CaO, CuO, Fe_2_O_3_, and Os with a mole ratio of 1:3:1:2 were used as starting materials. CaO was produced by sintering CaCO_3_ at 1273 K for 10 h in an atmosphere of Ar gas. An appropriate amount of KClO_4_ was added as the oxidizing agent. The raw materials were thoroughly mixed and ground using an agate mortar in a glove box filled with Ar gas. Then, the mixture was pressed into a platinum capsule for HPHT synthesis. A cubic-anvil-type high-pressure apparatus was adopted for the HPHT experiment. For the CCFOO-ordered compound, the pressure was slowly increased to 8 GPa, and the reactants were heated at 1373 K for 30 min. Then, the heating power was cut off, and the temperature was quickly dropped to room temperature in several seconds. After that, the pressure was slowly decreased to ambient within 8 h. For the CCFOO-disordered compound, the reactants were pressed to 6 GPa and heated at 1673 K for 20 min. Then, the heating power was cut off, and the pressure was slowly released to ambient within 6 h. Finally, the CCFOO-ordered and CCFOO-disordered products were grained and then rinsed with deionized water to wipe off the residual KCl.

Powder X-ray diffraction (XRD) was carried out on a Huber X-ray diffractometer (40 kV, 30 mA) with Cu-*K*_α1_ radiation. The scanning 2θ angle range was from 10 to 100°, with a step size of 0.005°. The crystallographic parameters were refined using the GSAS package.^22^ A small amount of impurities (∼3% in weight) was excluded in the refinement. The XAS spectra at the Cu-*L*_3_ and Fe-*L*_2,3_ edges were measured at beamline TLS11A of NSRRC, and the XAS spectrum at the Os-*L*_3_ edge was measured at beamline TLS17C of NSRRC. The XMCD patterns at the Cu-*L*_2,3_ and Fe-*L*_2,3_ edges were collected under 4.2 K and 6 T at beamline DEIMOS of synchrotron SOLEIL. The XMCD patterns at the Os-*L*_2,3_ edge were collected under 300 K and 7 T at beamline ID12 of the European synchrotron radiation facility (ESRF). Both the direction of the applied magnetic field and the helicity of X-rays (∼97%) were alternately flipped after each energy scan to obtain the μ^+^ (parallel) and μ^–^ (antiparallel) spectra.

The magnetic susceptibility and isothermal magnetization were measured using a Quantum Design superconducting quantum interference device magnetometer (MPMS-3). Both zero-field-cooling (ZFC) and field-cooling (FC) modes were adopted for magnetic susceptibility measurements at 0.1 T. The resistivity was measured using a pellet with a size of about 2 × 1 × 1 mm^3^ by a standard four-probe method on a Quantum Design physical property measurement system (PPMS-7). The heat capacity was measured using a bulk with a size of about 2 × 2 × 0.4 mm^3^ on PPMS-7.

## 3 Results and Discussion

**Figure 2:**
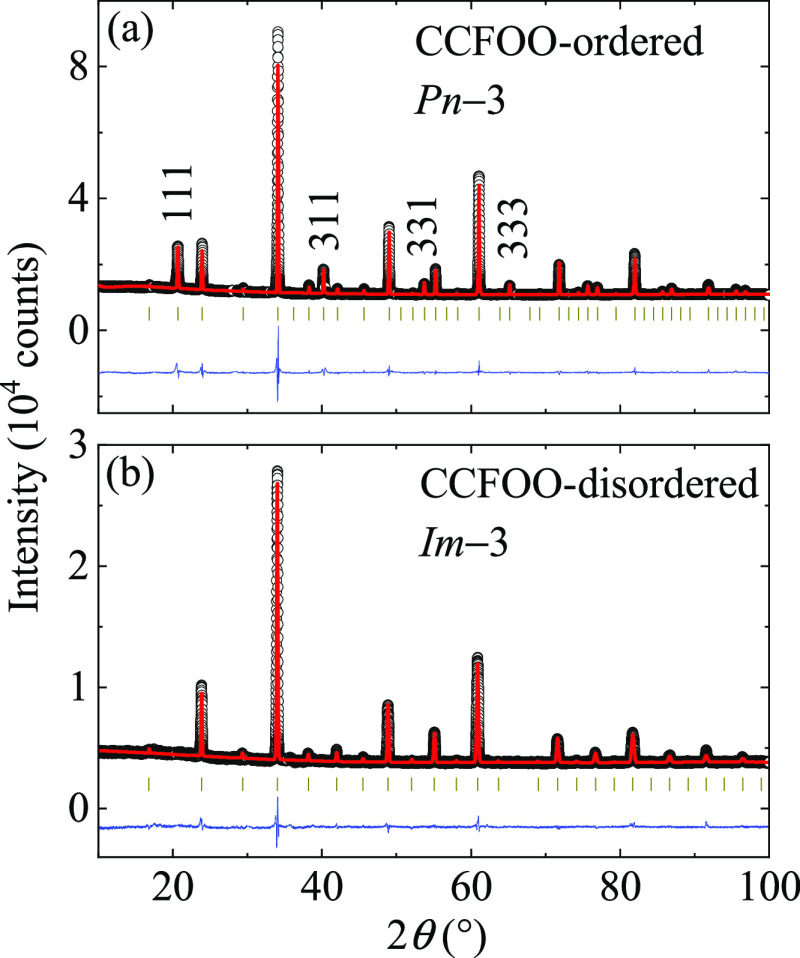
XRD patterns of (a) CCFOO-ordered and (b) CCFOO-disordered. The black circles, red lines, and blue lines indicate the observed, calculated, and difference, respectively. The ticks indicate the allowed Bragg reflections for the related space groups.

Figure 2a,b shows the XRD patterns as well as the refined plots of CCFOO-ordered and CCFOO-disordered, respectively. A series of peaks with *h* + *k* + *l* = odd, such as (111) and (311), can be clearly found in the XRD pattern of CCFOO-ordered (Figure 2a), strongly indicating the formation of the ordered B/B′-site in the compound. In sharp contrast, these characteristic peaks are absent in the XRD pattern of CCFOO-disordered (Figure 2b). Via the Rietveld analysis, we determine that CCFOO-ordered crystallizes to a *Pn*3̅ space group (No. 201), indicating the 1:3 ordered distribution of Ca and Cu at the A- and A′-site and the rock-salt-type distribution of Fe and Os at the B- and B′-sites, respectively. Note that during the refinement, a 9% antisite occupation was found for the B-site Fe and B′-site Os, similar to a previous study (11%).^11^ In comparison, CCFOO-disordered crystallizes to an *Im*3̅ space group (No. 204), indicating the disordered distribution of Fe and Os at the B-site. It is worth noting that if we refine CCFOO-disordered using a B-site ordered *Pn*3̅ space group, a nearly 50% antisite of Fe and Os is found to occur, suggesting a totally disordered B-site distribution. Thus, using the same reactants under different HPHT procedures, two kinds of quadruple perovskite oxides CCFOO with B-site ordering and disordering can be obtained. The refined parameters for CCFOO-ordered and CCFOO-disordered are listed in Table 1. Based on the refined bond lengths, the bond valence sum (BVS) calculations^23,24^ give the valence states of Ca^2+^ and Cu^2+^ for both CCFOO compounds. For CCFOO-ordered, the valence states of Fe^3+^ and Os^5+^ can also be determined using BVS calculations. For CCFOO-disordered, BVS calculations cannot be meaningfully applied due to the uncertainties in the bond lengths.

**Table 1:** Structural Parameters of B-Site Ordered and Disordered CCFOO^a^

parameters	CCFOO-ordered	parameters	CCFOO-disordered
*a* (Å)	7.4345(1)	*a* (Å)	7.446(10)
*x* (O)	0.2582(6)	*y* (O)	0.3099(7)
*y* (O)	0.4239(9)	*z* (O)	0.1797(7)
*z* (O)	0.5568(10)	*U*_iso_ (Ca) (100 × Å^2^)	0.197(7)
*G* (4*b* for Fe_1_)	0.907(2)	*U*_iso_ (Cu) (100 × Å^2^)	0.013(1)
*G* (4*b* for Os_1_)	0.093(2)	*U*_iso_ (Fe) (100 × Å^2^)	0.006(1)
*G* (4*c* for Fe_2_)	0.093(2)	*U*_iso_ (Os) (100 × Å^2^)	0.009(1)
*G* (4*c* for Os_2_)	0.907(2)	*U*_iso_ (O) (100 × Å^2^)	0.046(2)
*U*_iso_ (Ca) (100 × Å^2^)	0.011(2)	*d*_Ca-O_ (Å) (×12)	2.668(7)
*U*_iso_ (Cu) (100 × Å^2^)	0.012(1)	*d*_Cu-O_ (Å) (×4)	1.948(5)
*U*_iso_ (Fe_1_) (100 × Å^2^)	0.003(1)	*d*_Fe/Os-O_ (Å) (×6)	1.985(3)
*U*_iso_ (Os_2_) (100 × Å^2^)	0.013(1)	∠Fe/Os-O-Fe/Os (°)	139.44(21)
*U*_iso_ (O) (100 × Å^2^)	0.007(1)	BVS (Ca)	1.80
*d*_Ca-O_ (Å) (×12)	2.622(9)	BVS (Cu)	1.93
*d*_Cu-O_ (Å) (×4)	1.934(4)	*R*_wp_ (%)	4.50
*d*_Fe-O_ (Å) (×6)	2.045(4)	*R*_p_ (%)	3.27
*d*_Os-O_ (Å) (×6)	1.931(4)		
∠Fe_1_-O-Os_2_ (°)	138.39(22)		
BVS (Ca)	2.04		
BVS (Cu)	2.01		
BVS (Fe)	2.82		
BVS (Os)	5.06		
*R*_wp_ (%)	5.81		
*R*_p_ (%)	3.54		

a For CCFOO-ordered, the space group is *Pn*3̅ (No. 201) and the Wyckoff positions are Ca 2*a* (0.25, 0.25, 0.25), Cu 6*d* (0.25, 0.75, 0.75), Fe 4*b* (0, 0, 0), Os 4*c* (0.5, 0.5, 0.5), and O 24*h* (*x*, *y*, *z*). For CCFOO-disordered, the space group is *Im*3̅ (No. 204) and the Wyckoff positions are Ca 2*a* (0, 0, 0), Cu 6*b* (0, 0.5, 0.5), Fe/Os 8*c* (0.25, 0.25, 0.25), and O 24*g* (0, *y*, *z*). The BVS values (*V_i_*) were calculated using the formula *V_i_* = ∑_*j*_*S*_*ij*_, and *S_ij_* = exp[(*r*_0_ – *r*_ij_)/0.37]. The values of *r*_0_ are 1.967 for Ca, 1.679 for Cu, 1.765 for Fe, and 1.868 for Os. The parameter *G* represents site occupancy.

**Figure 3:**
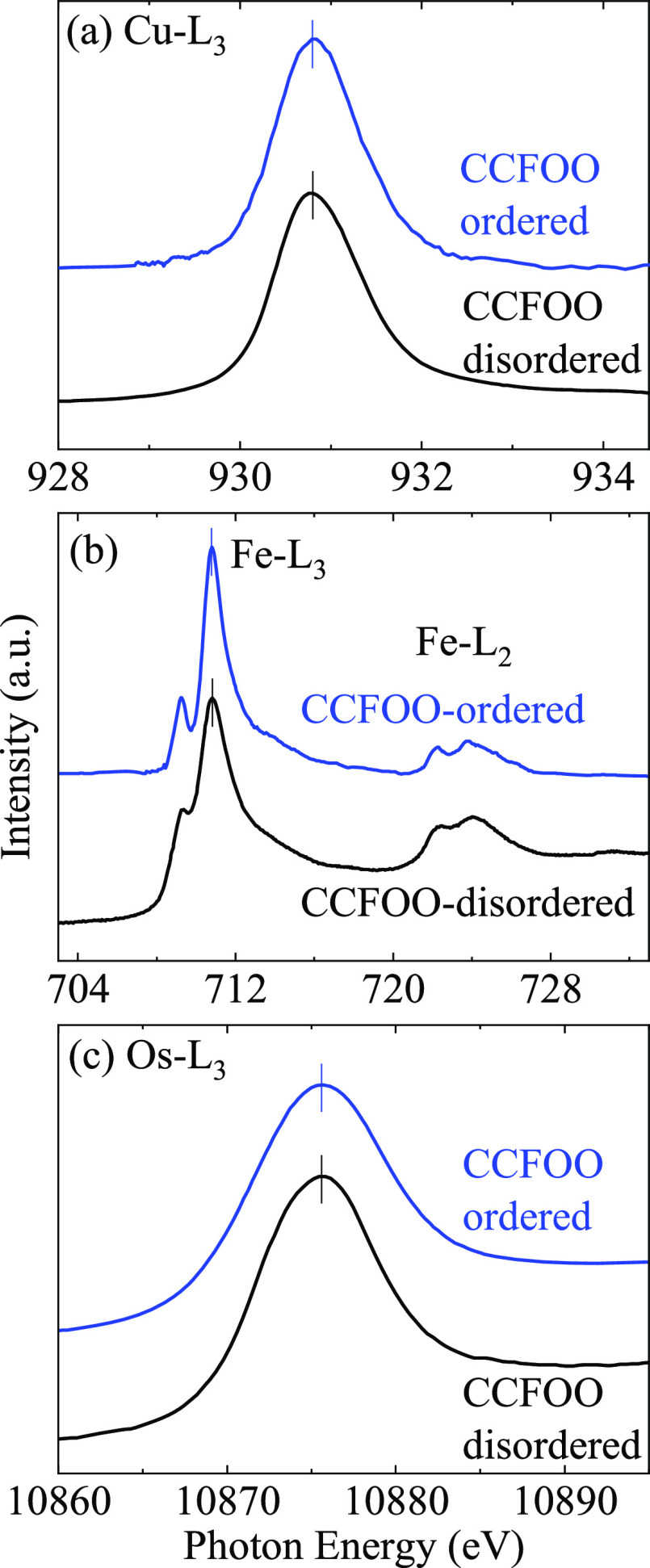
XAS spectra at the (a) Cu-*L*_3_, (b) Fe-*L*_2,3_, and (c) Os-*L*_3_ edges of CCFOO-ordered and CCFOO-disordered. The ticks indicate the position of the XAS peaks.

To directly obtain the valence states of the transition metal ions, especially of the CCFOO-disordered compound, we performed XAS measurements on both CCFOO compounds. It is well known that element-selective XAS at the transition metal *L*_2,3_ edges is highly sensitive to the valence state. For an open 3*d* shell system, an increase in the valence of the transition metal ion by one leads to a shift of the *L*_2,3_ XAS spectrum to the higher energies by one eV or more, accompanied by remarkable changes in the spectral feature.^25−27^ On account of its element-selective feature, XAS is especially appropriate for studying complex, multielement, and disordered systems such as our CCFOO. As shown in Figure 3a, the Cu-*L*_3_ XAS peaks of both CCFOO compounds locate at the same energy position (930.8 eV), indicating Cu^2+^ in both compounds. One should note that both of the Cu-*L*_3_ peaks locate at 0.5 eV lower in energy than that of CuO (931.3 eV).^28,29^ This energy shift comes from the modest crystal field of Cu in CCFOO (CuO_4_ square-planar) compared with that of CuO (CuO_6_ octahedron). The Fe-*L*_2,3_ XAS spectra are displayed in Figure 3b. It is clear that the main peak locates at the same energy for both CCFOO compounds, indicating the Fe^3+^ valence state, whereas the disorder leads mainly to somewhat broader spectral features. Figure 3c displays the Os-*L*_3_ XAS spectra. The Os-*L*_3_ XAS peaks for both CCFOO compounds also locate at the identical energy position, also indicating the same valence state of Os^5+^ in both CCFOO compounds. Hereto, we can conclude that the valence states scheme of both CCFOO-ordered and CCFOO-disordered to be Cu^2+^Fe^3+^Os^5+^. This result for CCFOO-ordered is in accordance with the BVS analysis, while for CCFOO-disordered, as explained above, BVS was not capable of providing the numbers.

**Figure 4:**
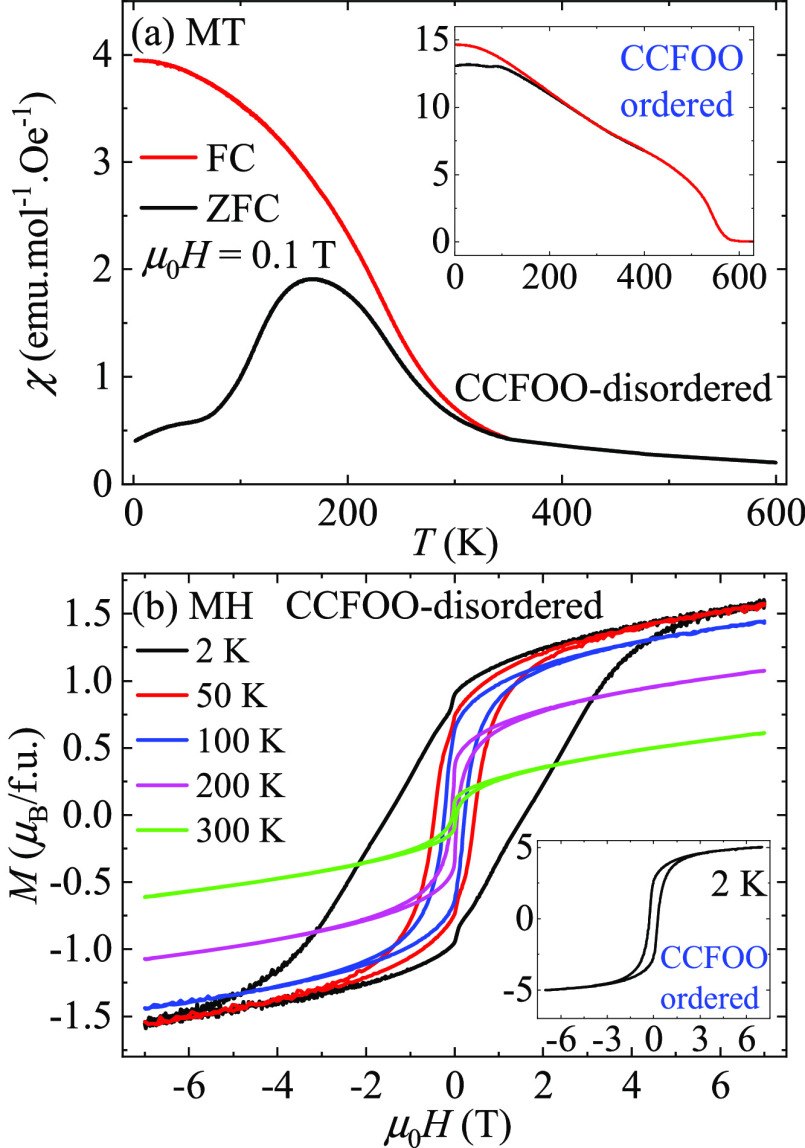
(a) Temperature-dependent susceptibility of CCFOO-disordered and CCFOO-ordered (inset). (b) Field-dependent magnetization of CCFOO-disordered at selected temperatures. The inset displays the magnetization of CCFOO-ordered at 2 K.

Figure 4a displays the magnetic susceptibility of CCFOO-disordered. Compared with the high *T*_C_ (580 K) and the sharp transition of CCFOO-ordered (inset of Figure 4a and ref (11)), the magnetic transition of CCFOO-disordered occurs at a lower temperature of 350 K and exhibits a quite moderate feature, effectively indicating a reduced magnetic coupling. One can also observe that the ZFC curve separates from the FC curve below 350 K and experiences a drop after its maximum at about 160 K. These features indicate spin-glass behavior due to frustrated magnetic interactions. Figure 4b displays the isothermal magnetization of CCFOO-disordered measured at selected temperatures. One can find a large coercive field of 2 T at 2 K. At high fields of up to 7 T, the magnetization (1.6 μ_B_/fu) is still reluctant to saturate and increases monotonically with increasing field, also indicating a strongly frustrated magnetic structure in CCFOO-disordered. For comparison, the magnetization of CCFOO-ordered behaves as a canonical long-range ferro-/ferrimagnet, with a much smaller coercive field (0.25 T) and a much larger saturated magnetization (5.0 μ_B_/fu), as shown in the inset of Figure 4b.^11^

It can be expected that disorder decreases the magnetic ordering temperature. For the double perovskite La_2_MnCoO_6_, the Mn/Co disorder leads to a lowering of the ordering temperature from 225 to 150 K.^18^ Interestingly, the valence states of both the Mn and Co ions are different between the ordered and disordered materials, and the presence of nonmagnetic low-spin Co^3+^ in the disordered sample is the main reason for the reduced magnetic ordering temperature. For the quadruple perovskite CaCu_3_Fe_2_Nb_2_O_12_, with Cu and Fe forming a FiM structure, the magnetic transition temperature of the disordered sample decreases as a result of the presence of antiphase boundaries.^13^ Our CCFOO system is different. The valence configuration remains the same for CCFOO-ordered and CCFOO-disordered.

**Figure 5:**
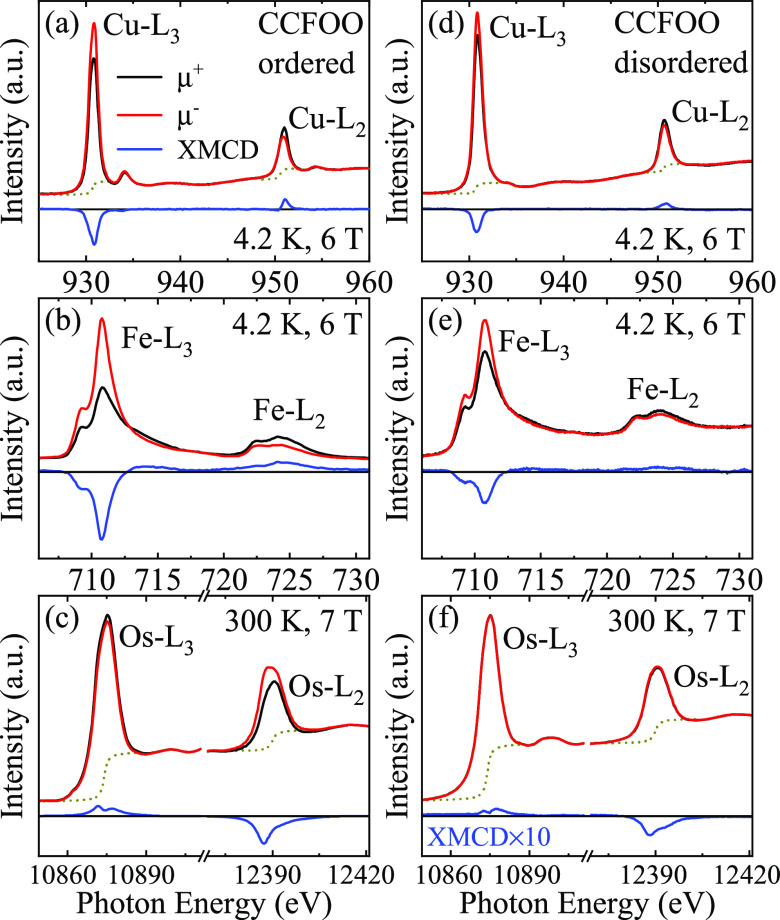
XMCD spectra at the (a) Cu-*L*_2,3_, (b) Fe-*L*_2,3_, and (c) Os-*L*_2,3_ edges of CCFOO-ordered. XMCD at the (d) Cu-*L*_2,3_, (e) Fe-*L*_2,3_, and (f) Os-*L*_2,3_ edges of CCFOO-disordered. The XAS with light polarization parallel (μ^+^, black lines) and antiparallel (μ^–^, red lines) with the magnetic field are shown. The blue lines are the XMCD (μ^+^ – μ^–^) spectra. The dashed lines indicate the edge jump.

To get a deeper insight into the magnetic properties, we performed XMCD measurements of both CCFOO-ordered and CCFOO-disordered compounds. Figure 5a,b reproduces the Cu-*L*_2,3_ and the Fe-*L*_2,3_ XMCD spectra of CCFOO-ordered, respectively, from ref (11). Figure 5c displays the Os-*L*_2,3_ XMCD spectrum measured in this study. The size of the XMCD signal [defined as (μ^+^ – μ^–^)/(μ^+^ + μ^–^)] at the Os-*L*_2_ edge reaches 24%, which is the largest value at room temperature for Os^5+^ compounds reported so far.^3,12,30−36^ One can observe that the *L*_3_ (*L*_2_) edges of Cu and Fe are negative (positive), opposite to that of the Os. Thus, our XMCD measurements experimentally confirm the Cu^2+^(↑)Fe^3+^(↑)Os^5+^(↓) magnetic arrangement.^11^

The XMCD spectra of CCFOO-disordered are shown in Figure 5d–f. Here, one can notice that the XMCD amplitudes decrease for all three transition metal constituents, Cu (Figure 5d), Fe (Figure 5e), and Os (Figure 5f), as compared to those in CCFOO-ordered (Figure 5a–c). Yet, one can clearly observe that the spin of the Os is antiparallel to those of the Cu and Fe. We can, therefore, safely conclude that CCFOO-disordered has the same Cu^2+^(↑)Fe^3+^(↑)Os^5+^(↓) magnetic arrangement as CCFOO-ordered. Here, we note that the Os XMCD signal in CCFOO-disordered has decreased significantly as compared to that in CCFOO-ordered. This is due to the fact that the Os XMCD has been performed at 300 K, which is only 50 K below the magnetic ordering temperature of CCFOO-disordered (350 K) but 280 K below the ordering temperature of CCFOO-ordered (580 K); see also Figure 4a for comparison.

**Figure 6:**
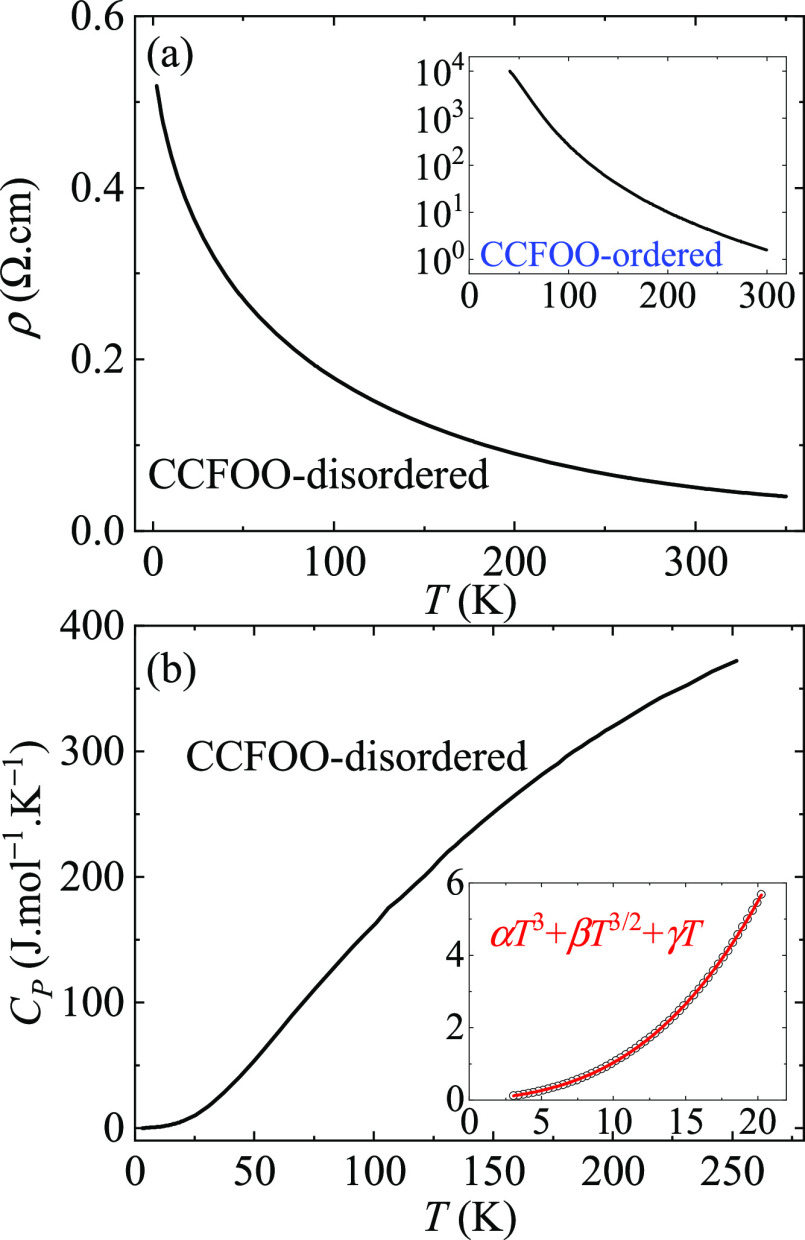
(a) Temperature-dependent resistivity of CCFOO-disordered and CCFOO-ordered (inset). (b) Temperature-dependent heat capacity of CCFOO-disordered. The inset displays the fitting below 20 K with the formula *C*_P_ = α*T*^3^ + β*T*^3/2^ + γ*T*. The black circles represent the measured data, and the red curve is the fitting result.

Finally, we investigated the electric properties. CCFOO-ordered has been reported to be an insulator with a band gap of 1.0 eV. The insulating behavior is stabilized by the ordering of the B-site Fe and B′-site Os.^11^ From this point of view, for CCFOO-disordered, with the Fe and Os randomly distributed at the B-sites, one may expect to find a more conductive behavior. Figure 6a depicts the temperature-dependent resistivity of CCFOO-disordered. Indeed, the resistivity is much lower than that for the ordered compound. The resistivity at 2 K is a modest ∼0.5 Ω cm for CCFOO-disordered. We note that similar *R*–*T* behavior can be observed in more metallic perovskite oxides.^3,12,37,38^ We further measured the heat capacity for CCFOO-disordered, as shown in Figure 6b. The plot can be well fitted with the formula *C*_P_ = α*T*^3^ + β*T*^3/2^ + γ*T* below 20 K with α = 5.1 × 10^–4^ J mol^–1^ K^–4^, β = 1.4 × 10^–2^ J mol^–1^ K^–5/2^, and γ = 8.1 × 10^–3^ J mol^–1^ K^–2^. Obviously, the presence of the γ term indicates the conductive nature of CCFOO-disordered.

## 4 Conclusions

In summary, under different HPHT conditions, we successfully synthesized the B-site Fe/Os ordered and disordered quadruple perovskite oxides CaCu_3_Fe_2_Os_2_O_12_. CCFOO-ordered crystallizes to a *Pn*3̅ space group, in which the B-site Fe and Os are orderly distributed in a rock-salt-type fashion. Using XAS and XMCD, we confirmed the Cu^2+^(↑)Fe^3+^(↑)Os^5+^(↓) valence and magnetic arrangement. CCFOO-disordered, on the other hand, crystallizes into an *Im*3̅ space group, where Fe and Os disorderly occupy the B-site. XAS revealed the Cu^2+^, Fe^3+^, and Os^5+^ valence states, which are the same as those of the B-site ordered counterpart. Although XMCD showed smaller effective local moments, which is consistent with the lower magnetic ordering temperature, the average spin arrangement is still that of the ordered compound. The relative robustness of the magnetic properties against disorder may be taken as an indication of the presence of short-range order of the B-site cations.
